# The issue of plasma asymmetric dimethylarginine reference range – A systematic review and meta-analysis

**DOI:** 10.1371/journal.pone.0177493

**Published:** 2017-05-11

**Authors:** Balázs Németh, Zénó Ajtay, László Hejjel, Tamás Ferenci, Zoltán Ábrám, Edit Murányi, István Kiss

**Affiliations:** 1 Department of Public Health Medicine, Medical School, University of Pécs, Pécs, Hungary; 2 Heart Institute, Medical School, University of Pécs, Pécs, Hungary; 3 John von Neumann Faculty of Informatics, Physiological Controls Group, Óbudai University, Budapest, Hungary; 4 Department of Hygiene, University of Medicine and Pharmacy from Tirgu Mures, Tirgu Mures, Romania; University of Adelaide, AUSTRALIA

## Abstract

**Background:**

Asymmetric dimethylarginine (ADMA) is an endogenous inhibitor of nitric oxide synthase, marker and mediator of endothelial dysfunction. Several studies have demonstrated its value in cardiovascular risk stratification and all-cause mortality prediction. The aim was to determine the reference range of plasma ADMA in healthy adults.

**Methods and results:**

Taking into account the most widely used ADMA measurement methods, only studies using either high performance liquid chromatography (HPLC) -with fluorescence or mass spectrometric detection-, or enzyme-linked immunosorbent assay (ELISA) to quantify plasma ADMA concentrations were enrolled. 66 studies were included in the quantitative analysis (24 using ELISA and 42 using HPLC) reporting a total number of 5528 non-diabetic, non-hypertensive, non-obese adults without any medication (3178 men and 2350 women, 41.6 ± 16.9 years old). The reference range of ADMA (in μmol/l with 95% confidence interval in parenthesis) was 0.34 (0.29–0.38)– 1.10 (0.85–1.35) with a mean of 0.71 (0.57–0.85) (n = 4093) measured by HPLC and 0.25 (0.18–0.31)– 0.92 (0.76–1.09) with a mean of 0.57 (0.48–0.66) (n = 1435) by ELISA.

**Conclusions:**

Numerous publications suggested that asymmetric dimethylarginine is not only an outstanding tool of disease outcome prediction but also a new potential therapeutic target substance; the reference range provided by this meta-analysis can become of great importance and aid to further investigations. However, developing a standard measurement method would be beneficial to facilitate the clinical usage of ADMA.

## Introduction

Asymmetric dimethylarginine (ADMA) is a naturally occurring substance produced as a by-product of the proteolysis of post-translational methylated proteins [1]. Its major function is the competitive inhibition of nitric oxide synthase (NOS), which is responsible for generating nitric oxide (NO) from L-Arginine (L-Arg) [2]. Approximately 15% of the generated ADMA is excreted through the renal system. The remaining amount is degraded by the dimethylarginine dimethyl aminohydrolase (DDAH) enzyme, which is impaired by oxidative stress [3]. Moreover, ADMA has been shown capable of uncoupling electron transport between L-Arg and NOS resulting in production of reactive oxygen species [4]. Accordingly, ADMA can be a useful marker and mediator of oxidative stress [1, 5]. Compared to healthy controls elevated ADMA concentrations were found in patients suffering from hypertension, coronary artery disease (CAD), heart failure, stroke, obesity, diabetes mellitus, kidney injury and even inflammatory bowel diseases, which are of high public health significance [6–11]. Correlation between intima media thickness and ADMA concentrations was also demonstrated [10, 11].

Multiple studies have found association between higher levels of ADMA and increased cardiovascular risk especially in the cases of CAD [12–14]. In a population-based cohort with a follow-up duration of 24 years, incidence of myocardial infarction and stroke increased with ADMA [15]. Furthermore, higher all-cause mortality was associated with elevated levels of ADMA in an 11-years-long prospective study of 3320 Framingham Offspring patients [16]. A recent meta-analysis involving nearly 20,000 patients has confirmed these results by showing a 1.42 risk ratio of adverse cardiovascular disease outcomes comparing ADMA values of the top tertile with the bottom tertile [17].

Currently, high performance liquid chromatography (HPLC), with fluorescence or mass spectrometric detection-, and enzyme-linked immunosorbent assay (ELISA) are the most frequently used analytical methods to determine plasma ADMA concentrations. HPLC is capable of determining the quantity of multiple substances at the same time (e.g. symmetric dimethylarginine, L-Arg), however its high maintenance cost and time-consuming analysis can be inconvenient. ELISA is an easy-to-use, fast method with the disadvantage of less sensitivity. Regarding the comparability of these methods, in case of the analysis of the same samples Schulze et al found nearly identical ADMA levels measured by ELISA and HPLC, while in another study Shiroka et al found ELISA to overestimate ADMA values compared to HPLC[18, 19]. Several studies aimed to determine the reference range of plasma ADMA, regrettably only few of them investigated completely healthy individuals (Table 1).

**Table 1:** Reference intervals of plasma ADMA.

HPLC			ELISA		
Author, date of publication	No. of participants	ADMA in μmol/L (2.5–97.5 percentiles)	Author, date of publication	No. of participants	ADMA in μmol/L (2.5 and 97.5 percentiles)
Blackwell, 2007 [3]	100	0.29–0.63	Deneva, 2011 [22]	150	0.22–0.69
Hov, 2007 [20]	238	0.40–0.77	Schultze, 2005 [23]	500	0.36–1.17
Schwedhelm, 2009 [21]	1124	0.311–0.732			

Here we report a systematic review and a meta-analysis conducted to determine the reference range of plasma ADMA in healthy adults.

## Materials and methods

### Data sources

This study was designed in conformity with the guidelines of the 2009 Preferred Reporting Items for Systematic Reviews and Meta-Analysis (PRISMA) statement.

On June 30th 2016 a comprehensive literature search was performed in Medline and Web of Science using the following keywords: “asymmetric dimethylarginine” AND “healthy” NOT “animal”. Literature search and managing of references were performed using “EndNote X7 software” (Thomson Reuters Crp. 3 Times Square, New York, New York, United States).

### Study selection

To be included in full text evaluation records had to: 1, report plasma ADMA concentrations; 2, report ADMA concentrations of healthy individuals; 3, report the method of ADMA analysis; 4, report≥20 patients. Review articles, meta-analyses and measurement methodical studies, were excluded. To analyze the data of an adult population papers reporting ADMA concentrations from individuals under the age of 18 were dropped. Furthermore, due to well-known endocrinological changes, studies investigating pregnant individuals were excluded.

After full text evaluation, to be included in quantitative analysis articles had to: 1, report ADMA values measured by either ELISA or HPLC; 2, report ADMA concentrations numerically; 3, state and/or indicate in “patients characteristics” that the controls are healthy individuals; 4, refer or report the method of ADMA measurement in detail.

Before statistical analysis another detailed review was performed to reveal diseases (e.g. hypertension, diabetes, obesity, etc.) and sample origin. Papers reporting any unhealthy individuals or other samples than plasma (e.g. serum, urine, cell cultures, ect.) were excluded from the final database.

Database search, abstract screening and full text evaluation were performed by two reviewers independently (BN and EM). In the case of shortcomings the corresponding author of the paper in question was contacted via e-mail. If the corresponding author was unavailable or unable to answer the manuscript was excluded. The final database contained the following parameters: name of the first author, publication date, ADMA levels in μmol/L, number of participants, age of participants, gender distribution, the applied method, percentage of smokers, country and region of the study, SBP, DBP and BMI. Continuous variables were recorded as mean + standard deviation (SD) or standard error of mean (SEM) or median + interquartile range (IQR). Due to the aims and the characteristics of this meta-analysis study quality assessment was not performed, in order to rule out potential bias.

### Quantitative data synthesis and analysis

Normal approximation was used for the meta-analysis, both for the mean and for the reference interval.

For studies where only median and IQR or median and minimum/maximum were given, the mean and SD were approximated using the method of Wan et al. [24]. Two studies reported 2.5–97.5% percentile, but no other details were provided. These studies were excluded from further analysis, as no meaningful estimation of mean and SD could be gathered from these data. Likewise, one study was excluded because it reported mean and median without any metric of dispersion. Confidence interval for the mean was calculated as mean ± 1.96·SD/n^1/2^, and was visualized with diamonds.

Reference interval was calculated as mean ± 1.96·SD (under the assumption of normality, this has a coverage of 95%). Confidence interval for the endpoints of the reference interval was also calculated with normal approximation [25]. In short, the mean and the variance are independent for normal distribution, thus the variance of mean ± 1.96·SD is the variance of mean ± 1.96^2^ times the variance of SD. The former can be estimated as variance/n^1/2^ (as sample variance is a consistent estimator of the population variance). As far as the latter is concerned, SD follows a χ-distribution (as variance follows a χ^2^-distribution) after appropriate scaling, namely $\frac{\left(n-1\right)}{\sigma^{2}}SD^{2}\sim \chi_{n-1}^{2}$, thus the variance of SD can be approximated as $\frac{SD^{2}}{\left(n-1\right)}D^{2}\left(\chi_{n-1}\right)=\frac{SD^{2}}{\left(n-1\right)}\cdot \left[n-1-2{\left(\frac{\Gamma \left(\frac{n}{2}\right)}{\Gamma \left(\frac{n-1}{2}\right)}\right)}^{2}\right]=SD^{2}\cdot \left[1-\frac{2}{n-1}{\left(\frac{\Gamma \left(\frac{n}{2}\right)}{\Gamma \left(\frac{n-1}{2}\right)}\right)}^{2}\right]$. Confidence interval was then constructed using the quantiles of *t*-distribution with *n-1* degrees of freedom (truncated at zero if it were negative); it will be visualized as shaded areas around the endpoints of the reference interval.

Normality can be very roughly assessed—without detailed distribution data—by comparing mean with median (where both was given) and median with the midpoint of IQR (when those were given). For our data, means and medians were very similar (IQR for the relative difference: 0.68%–5.68%), and also the IQR was rather symmetric around the median (IQR for the relative difference between median and the midpoint of IQR: (-3.55%)–(-1.00%)). Very few studies reported both SD and IQR, so they were not contrasted. Nevertheless, the available data indicates that normality is plausible. Also, no study had less than 20 participants to allow for central limit theorem to become effective (on one level higher, for the means).

Both fixed effects and random effects models were estimated, but due to the extreme heterogeneity, only the results of the random effects models are presented. The models were estimated using restricted maximum likelihood [26].

Effects of moderator variables were studied with standard meta-regression approach [26].

All calculations were performed under R statistical program package version 3.3.2 [27] with the metafor library version 1.9–9 [28] using a custom script that is available from the corresponding author on request.

## Results

**Fig 1:**
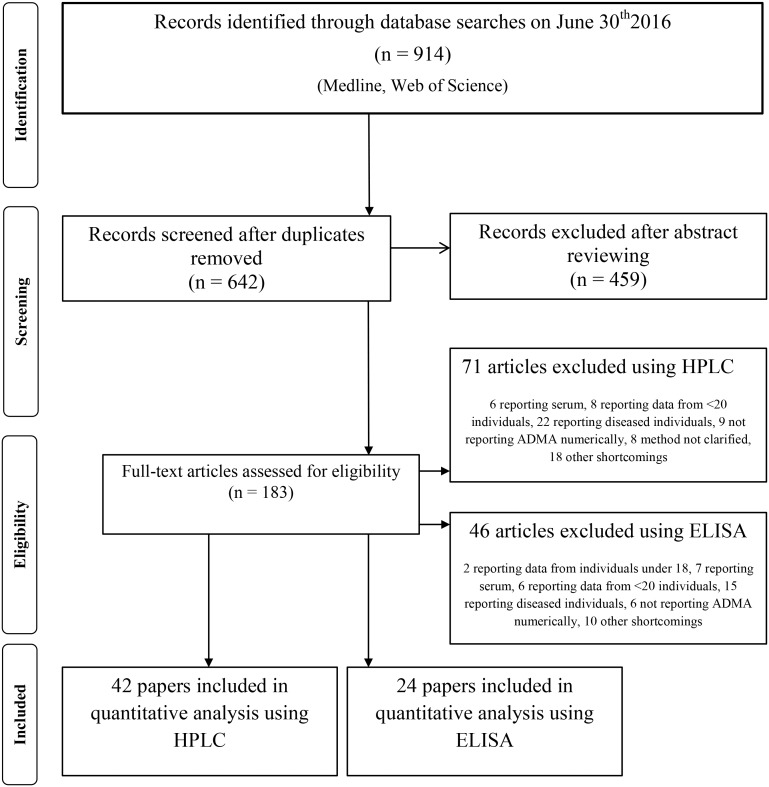
Study flow diagram. High performance liquid chromatography (HPLC), Enzyme-linked immunosorbent assay (ELISA), Asymmetric dimethylarginine (ADMA).

Using the method discussed above 914 citations were identified on June 30^th^ 2016. After dropping duplicates 642 abstracts were reviewed. After abstract screening, 183 records were included in full text evaluation. Eventually, 66 studies were included in the quantitative analysis (24 using ELISA and 44 using HPLC). Study selection process and detailed reasons of exclusions are indicated in Fig 1.

The detailed list of studies included in quantitative analysis is provided in supplementary files.

The total number of healthy individuals identified was 5528 (3178 men and 2350 women, 41.6 ± 16.9 years old). The majority of the studies involved in our article were undertaken in Europe (HPLC: 78.7%, ELISA: 93.1%). Due to the character of this meta-analysis most of the involved articles are case-control studies.

42 studies using HPLC were included in the statistical analysis. 36 of these articles referred to a HPLC method using fluorescence detection, the remaining 6 studies used LC/MS detection to measure ADMA concentrations.

24 studies using ELISA were involved in the quantitative analysis. 11 used ELISA kits purchased from DLD Diagnostika GmbH (Hamburg, Germany), 12 used ELISA kits purchased from Immundiagnostik (Bensheim, Germany) the remaining 1 referred to a method developed by Schulze et al. [18].

**Fig 2:**
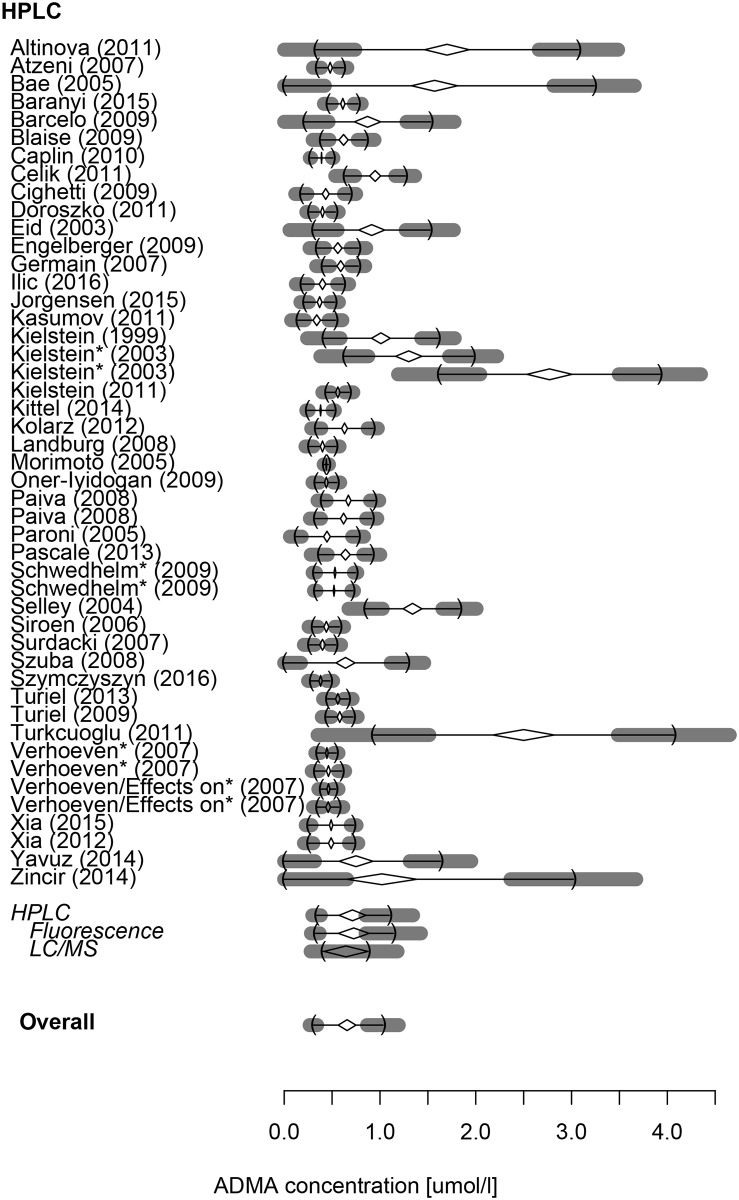
Forest plot detailing the reference intervals and means of plasma ADMA concentrations acquired from the involved studies using HPLC.

**Fig 3:**
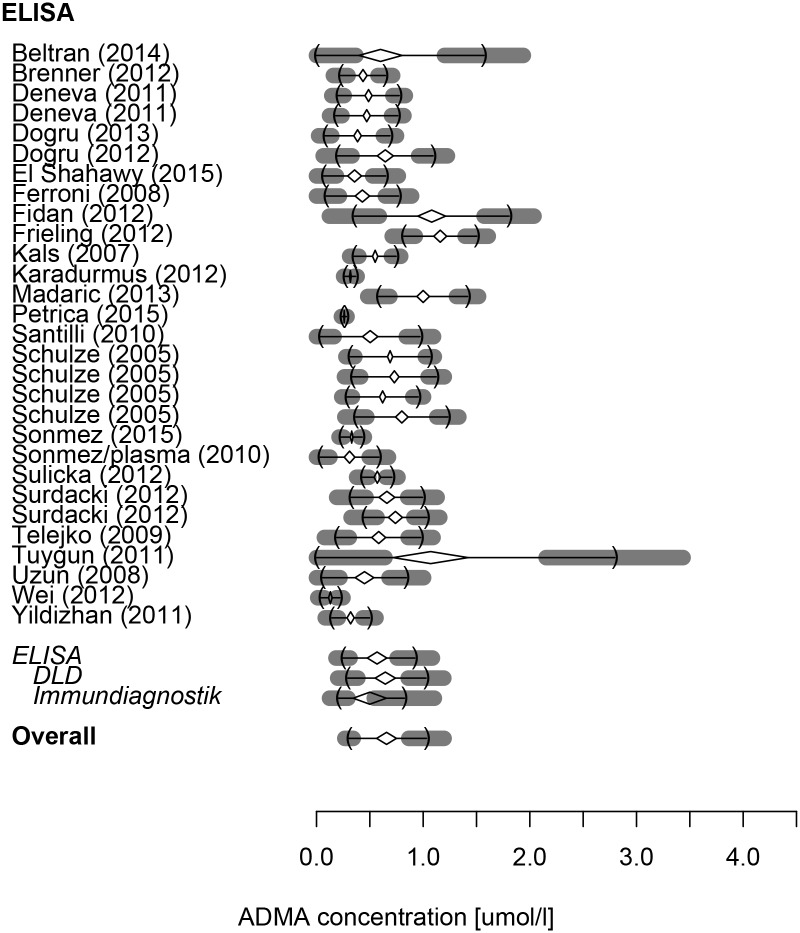
Forest plot detailing the reference intervals and means of plasma ADMA concentrations acquired from the involved studies using ELISA.

The reference range of ADMA (in μmol/l) was 0.34 (0.29–0.38)– 1.10 (0.85–1.35) with a mean of 0.71 (0.57–0.85) (n = 4093) measured by HPLC and 0.25 (0.18–0.31)– 0.92 (0.76–1.09) with a mean of 0.57 (0.48–0.66) (n = 1435) by ELISA. Detailed results for each subgroup are given in Table 2. Overall, with both methods combined, ADMA had a reference range of 0.30 (0.27–0.34)– 1.03 (0.87–1.20) (mean: 0.66 [0.56–0.75]). These results are visualized as forest plot on Figs 2 and 3.

**Table 2:** ADMA 95% reference intervals and means (with 95% confidence interval) according to subgroup analysis of method types.

	HPLC		ELISA^a^	
	Fluorescencen = 2852	LC/MSn = 1241	DLDn = 952	Immundiagnostikn = 455
**Reference interval**	0.32 (0.28–0.37)– 1.14 (0.85–1.43)	0.41 (0.28–0.53)– 0.87 (0.56–1.18)	0.29 (0.20–0.39)– 1.03 (0.86–1.19)	0.21 (0.12–0.65)– 0.82 (0.54–1.10)
**Mean**	0.73 (0.56–0.89)	0.64 (0.42–0.87)	0.65 (0.55–0.74)	0.50 (0.34–0.65)

^a^1 study using another method is not included

The overall results had extreme heterogeneity (Q = 16074, p<0.001; τ^2^ = 0.1682 (SE = 0.0279); I^2^ = 99.96%; H^2^ = 2438).

As possible moderator variables, average age, ratio of males, average BMI and type of analysis (HPLC or ELISA) were tested in meta-regression for mean ADMA. Overall, the moderator variables were jointly significant (QM = 11.38, p = 0.0226), but the only significant difference individually was the effect of BMI: studies with higher average BMI were associated with lower mean ADMA (0.1097 μmol/l decrease for each unit of increase in BMI, p = 0.0067). In particular, measurement method was not associated with different mean ADMA (p = 0.0866).

### Limitations

Some of the included publications provided limited methodological and anthropometric data. 58% were unable to provide details regarding the anticoagulant substance used in blood collection tubes, which could affect ADMA levels. Moreover, 36% were unable to provide any BMI data.

## Discussion

The results of this study are based on the plasma ADMA levels of 5528 apparently healthy individuals, which is eligible to calculate a proper reference interval. Most of the studies provided narrow mean ADMA concentrations with low standard deviation, which indicates that the involved subjects had similar ADMA levels. Seemingly, the number of participants suffering from ADMA altering conditions was low. Moreover, according to the meta-regression, age had no significant influence on ADMA levels, which can indicate that the involved individuals were indeed healthy. The study identification and selection method used in this manuscript were sufficient to enroll a population, which is appropriate for analysis.

In contrast with the results of Sydow et al., who investigated 980 healthy adults and found positive correlation between ADMA and BMI [6]; this meta-analysis showed a 0.1097 μmol/l decrease of ADMA for each unit of increase in BMI. This result is based on the data of 3233 individuals, because not every study was able to provide BMI values. Moreover, the average weighted standard deviation of the BMI was 2.69, which can be considered low. To the best of our knowledge, no previous studies including so many apparently healthy individuals were performed to investigate the connection of ADMA levels and BMI. Further research is needed to clarify the relation between ADMA and BMI among healthy individuals.

The aim of our study was to provide a proper reference interval for plasma ADMA. However, due to the high heterogeneity the interpretation of results can be challenging. The practical usage of the relatively wide reference interval determined by this meta-analysis is limited. This can be explained by pre-analytical errors, the usage of different laboratory equipment and evaluation softwares [29, 30], especially in the case of studies using HPLC. Some studies using HPLC provided noticeably higher ADMA levels. In the case of the study published by Zincir et al., the correct separation of asymmetric dimethylarginine from symmetric dimethylarginine can be doubted, however, ADMA and SDMA levels are presented separately [31]. Turkcuoglu et al. obtained nearly double plasma ADMA concentration as referred to in the methodological instructions of the kit that they have used; nevertheless, the possible reason for this is not discussed by the Authors [32]. Despite the fact that, these studies might have been biased by some methodological errors, they were not excluded because they were found eligible according to the criteria stated in materials and methods.

Interestingly, in the case of studies using ELISA, high heterogeneity persisted even in testing studies using ELISA kits developed by the same manufacturer.

Previous comparative studies found ELISA to overestimate plasma ADMA concentrations compared to HPLC [19,33,34].

In 2007 Horowitz et al. published a non-systematic overview, which included the plasma and serum ADMA levels of 2371 healthy individuals. Taking into account that the focus of this study is on the ADMA measurement methodological considerations, the comparability of mean plasma ADMA levels provided by this paper and the current meta-analysis is limited.

To the best of our knowledge, this is the first systematic review and meta-analysis investigating plasma ADMA levels in healthy individuals. Interestingly, the available data have shown opposite results regarding the plasma ADMA concentrations measured by HPLC and ELISA. Our study points out how analytical differences can result in almost incomparable results in the case of ADMA levels determined by HPLC and ELISA methods. Nevertheless, the results of this meta-analysis bring up several methodological questions connected to ADMA measurement, which could be answered by prominent researchers of the field.

### Study limitations

To get an appropriate population for a proper reference range, control groups containing any diseased individuals were excluded from the final analysis. Thus, if the authors were unable to provide a completely healthy group, relatively high numbers of individuals were lost because of a few diseased participants. Moreover, several studies were excluded due to minor shortcomings (e.g. showing different data in text and tables, not providing detailed measurement method or reference, not indicating sample origin precisely, possible misprints in measurement units), which were not corrected by the corresponding author upon questioning. It could also be addressed as limitation that due to the selection criteria all the relevant articles were included in the statistical analysis, thus a few papers with outlier data as well. However, the statistical evaluation performed without these outliers resulted in a negligible difference, thus the results of the whole dataset were published.

Furthermore, this meta-analysis found that the enrolled publications using ELISA provided lower ADMA concentrations compared to the papers using HPLC, which contradicts the results of previous comparative studies.

## Conclusion

Numerous publications suggest that ADMA is not only an outstanding tool of disease outcome prediction but also a new potential therapeutic target. According to the results of this meta-analysis developing a standard measurement method would be beneficial to facilitate the clinical usage of ADMA. Until then, clinical trials are recommended to enroll healthy controls.

## Supporting information

S1 Checklist PRISMA checklist. (DOC)

S1 Table List of papers included in quantitative analysis using HPLC to determine ADMA concentrations. (DOC)

S2 Table List of papers included in quantitative analysis using ELISA to determine ADMA concentrations. (DOC)

S3 Table Full datasheet. (XLSX)
