# Supplementary material for: The issue of plasma asymmetric dimethylarginine reference range – A systematic review and meta-analysis
Source: PLoS One. 2017 May 11;12(5):e0177493. doi: 10.1371/journal.pone.0177493 (PMC5426758; doi:10.1371/journal.pone.0177493)
Supplement: S1 Table — (DOC) [file pone.0177493.s002.doc]

| **Study** | **No. of participants** | **Method** | **Country** |
| --- | --- | --- | --- |
| Altinova 2007 (S1) | 35 | Fluorescence | Turkey, Europe |
| Atzeni 2011 (S2) | 35 | Fluorescence | UK, Italy, Europe |
| Bae 2005 (S3) | 48 | Fluorescence | South Korea |
| Baranyi 2015 (S4) | 48 | Fluorescence | Austria, Europe |
| Barcelo 2009 (S5) | 23 | Fluorescence | Spain, Europe |
| Blaise 2009 (S6) | 24 | LC/MS | France, Europe |
| Caplin 2010 (S7) | 822 | Fluorescence | UK, Europe |
| Celik 2011 (S8) | 30 | Fluorescence | Turkey,Europe |
| Cighetti 2009 (S9) | 50 | Fluorescence | Italy, Europe |
| Doroszko 2011 (S10) | 47 | Fluorescence | Poland, Europe |
| Eid 2003 (S11) | 20 | Fluorescence | Norway, Europe |
| Engelberger 2009 (S12) | 24 | LC/MS | Switzerland, Europe |
| Germain 2007 (S13) | 22 | Fluorescence | USA, North - America |
| Ilic 2016 (S14) | 38 | Fluorescence | Serbia, Europe |
| Jorgensen 2015 (S15) | 40 | Fluorescence | Denmark, Europe |
| Kasumov 2011 (S16) | 25 | Fluorescence | USA, North - America |
| Kielstein 1999 (S17) | 37 | Fluorescence | Germany, Europe |
| Kielstein 2003 (S18) | 48* | Fluorescence | Germany, Europe |
| Kielstein 2011 (S19) | 24 | LC/MS | Germany, Europe |
| Kittel 2014 (S20) | 400 | Fluorescence | Germany, Europe |
| Kolarz 2012 (S21) | 95 | Fluorescence | Poland, Europe |
| Landburg 2008 (S22) | 35 | Fluorescence | The Netherlands, Europe |
| Morimoto 2005 (S23) | 20 | Fluorescence | Japan, Asia |
| Oner-Iyidogan 2009 (S24) | 31 | Fluorescence | Turkey, Europe |
| Paiva 2008 (S25) | 179* | Fluorescence | Finland, Europe |
| Paroni 2005 (S26) | 85 | Fluorescence | Italy, Europe |
| Pascale 2013 (S27) | 31 | Fluorescence | Italy, Europe |
| Schwedhelm 2009 (S28) | 1124* | LC/MS | USA, North-America |
| Selley 2004 (S29) | 25 | LC/MS | Australia |
| Siroen 2006 (S30) | 27 | Fluorescence | The Netherlands, Europe |
| Surdacki 2007 (S31) | 20 | LC/MS | Poland, Europe |
| Szuba 2008 (S32) | 43 | Fluorescence | Poland, Europe |
| Szymczyszyn 2016 (S33) | 30 | Fluorescence | Poland, Europe |
| Turiel 2009 (S35) | 25 | Fluorescence | Italy, Europe |
| Turiel 2013 (S34) | 20 | Fluorescence | Italy, Europe |
| Turkcuoglu 2011 (S36) | 24 | Fluorescence | Turkey, Europe |
| Verhoeven 2007a (S37) | 90* | Fluorescence | The Netherlands, Europe |
| Verhoeven 2007b (S38) | 40* | Fluorescence | The Netherlands, Europe |
| Xia 2012 (S39) | 72 | Fluorescence | China, Asia |
| Xia 2015 (S40) | 182 | Fluorescence | China, Asia |
| Yavuz 2014 (S41) | 25 | Fluorescence | Turkey, Europe |
| Zincir 2014 (S42) | 30 | Fluorescence | Turkey, Europe |

**Table 3** List of papers included in quantitative analysis using HPLC to determine ADMA concentrations.

* Subgroup data available

S1) Altinova AE, Arslan M, Sepici-Dincel A, Akturk M, Altan N, Toruner FB. Uncomplicated type 1 diabetes is associated with increased asymmetric dimethylarginine concentrations. J Clin Endocrinol Metab. 2007; 92(5): 1881-1885.

S2) Atzeni F, Sarzi-Puttini P, Sitia S, Tomasoni L, Gianturco L, Battellino M, et al. Coronary flow reserve and asymmetric dimethylarginine levels: new measurements for identifying subclinical atherosclerosis in patients with psoriatic arthritis. J Rheumatol. 2011; 38(8): 1661-1664.

S3) Bae SW, Stuhlinger MC, Yoo HS, Yu KH, Park HK, Choi BY, et al. Plasma asymmetric dimethylarginine concentrations in newly diagnosed patients with acute myocardial infarction or unstable angina pectoris during two weeks of medical treatment. Am J Cardiol. 2005; 95(6): 729-733.

S4) Baranyi A, Amouzadeh-Ghadikolai O, Rothenhausler HB, Theokas S, Robier C, Baranyi M, et al. Nitric Oxide-Related Biological Pathways in Patients with Major Depression. PLoS One. 2015; 10(11): e0143397. doi: 10.1371/journal.pone.0143397

S5) Barcelo A, de la Pena M, Ayllon O, Vega-Agapito MV, Pierola J, Perez G, et al. Increased plasma levels of asymmetric dimethylarginine and soluble CD40 ligand in patients with sleep apnea. Respiration. 2009; 77(1): 85-90.

S6) Blaise S, Maas R, Trocme C, Kom GD, Roustit M, Carpentier PH, et al. Correlation of biomarkers of endothelium dysfunction and matrix remodeling in patients with systemic sclerosis. J Rheumatol. 2009; 36(5): 984-988.

S7) Caplin B, Nitsch D, Gill H, Hoefield R, Blackwell S, MacKenzie D, et al. Circulating methylarginine levels and the decline in renal function in patients with chronic kidney disease are modulated by DDAH1 polymorphisms. Kidney Int. 2010; 77(5): 459-467.

S8) Celik C, Cayci T, Ozdemir B, Akgul EO, Zincir S, Balikci A, et al. Plasma asymmetric dimethylarginine (ADMA) concentrations in patients with first and multiple episode schizophrenia. Psychiatry Res. 2011; 190(2-3): 177-180.

S9) Cighetti G, Fermo I, Aman CS, Ferraroni M, Secchi A, Fiorina P, et al. Dimethylarginines in complicated type 1 diabetes: roles of insulin, glucose, and oxidative stress. Free Radic Biol Med. 2009; 47(3): 307-311.

S10) Doroszko A, Andrzejak R, Szuba A. Role of the nitric oxide metabolic pathway and prostanoids in the pathogenesis of endothelial dysfunction and essential hypertension in young men. Hypertens Res. 2011; 34(1): 79-86, doi: 10.1038/hr.2010.169

S11) Eid HM, Eritsland J, Larsen J, Arnesen H, Seljeflot I. Increased levels of asymmetric dimethylarginine in populations at risk for atherosclerotic disease. Effects of pravastatin. Atherosclerosis. 2003; 166(2): 279-284.

S12) Engelberger RP, Teta D, Henry H, De Senarclens O, Dischl B, Liaudet L, et al. Haemodialysis acutely reduces the plasma levels of ADMA without reversing impaired NO-dependent vasodilation. Clin Sci. (Lond) 2009; 117(8): 293-303, doi: 10.1042/CS20080561

S13) Germain AM, Romanik MC, Guerra I, Solari S, Reyes MS, Johnson RJ, et al. Endothelial dysfunction - A link among preeclampsia, recurrent pregnancy loss, and future cardiovascular events? Hypertension. 2007; 49(1): 90-95, doi: 10.1161/01.Hyp.0000251522.18094.D4

S14) Ilic MD, Pavlovic R, Lazarevic G, Zivanovic S, Cvetkovic T, Kocic G, et al. Detrimental effects of a bout of physical exercise on circulating endogenous inhibitors of endothelial function in patients with coronary artery disease. J Cardiovasc Med. (Hagerstown) 2016, doi: 10.2459/JCM.0000000000000400

S15) Jorgensen A, Knorr U, Soendergaard MG, Lykkesfeldt J, Fink-Jensen A, Poulsen HE, et al. Asymmetric dimethylarginine in somatically healthy schizophrenia patients treated with atypical antipsychotics: a case-control study. BMC Psychiatry. 2015; 15: 67, doi: 10.1186/s12888-015-0455-4

S16) Kasumov T, Edmison JM, Dasarathy S, Bennett C, Lopez R, Kalhan SC. Plasma levels of asymmetric dimethylarginine in patients with biopsy-proven nonalcoholic fatty liver disease. Metabolism. 2011; 60(6): 776-781, doi: 10.1016/j.metabol.2010.07.027

S17) Kielstein JT, Boger RH, Bode-Boger SM, Schaffer J, Barbey M, Koch KM, et al. Asymmetric dimethylarginine plasma concentrations differ in patients with end-stage renal disease: relationship to treatment method and atherosclerotic disease. J Am Soc Nephrol. 1999; 10(3): 594-600.

S18) Kielstein JT, Bode-Boger SM, Frolich JC, Ritz E, Haller H, Fliser D. Asymmetric dimethylarginine, blood pressure, and renal perfusion in elderly subjects. Circulation. 2003; 107(14): 1891-1895, doi: 10.1161/01.CIR.0000060496.23144.A7

S19) Kielstein JT, Veldink H, Martens-Lobenhoffer J, Haller H, Perthel R, Lovric S, et al. Unilateral nephrectomy causes an abrupt increase in inflammatory mediators and a simultaneous decrease in plasma ADMA: a study in living kidney donors. Am J Physiol Renal Physiol. 2011; 301(5): F1042-1046. doi: 10.1152/ajprenal.00640.2010

S20) Kittel A, Muller F, Konig J, Mieth M, Sticht H, Zolk O, et al. Alanine-glyoxylate aminotransferase 2 (AGXT2) polymorphisms have considerable impact on methylarginine and beta-aminoisobutyrate metabolism in healthy volunteers. PLoS One. 2014; 9(2): e88544. doi: 10.1371/journal.pone.0088544

S21) Kolarz M, Glowacki R, Stompor T, Wyroslak J, Undas A. Elevated levels of Nepsilon-homocysteinyl-lysine isopeptide in patients on long-term hemodialysis. Clin Chem Lab Med. 2012; 50(8): 1373-1378, doi: 10.1515/cclm-2011-0716

S22) Landburg PP, Teerlink T, Muskiet FA, Duits AJ, Schnog JJ, Curama study g. Plasma concentrations of asymmetric dimethylarginine, an endogenous nitric oxide synthase inhibitor, are elevated in sickle cell patients but do not increase further during painful crisis. Am J Hematol. 2008; 83(7): 577-579, doi: 10.1002/ajh.21184

S23) Morimoto H, Nakao K, Fukuoka K, Sarai A, Yano A, Kihara T, et al. Long-term use of vitamin E-coated polysulfone membrane reduces oxidative stress markers in haemodialysis patients. Nephrol Dial Transplant. 2005; 20(12): 2775-2782, doi: 10.1093/ndt/gfi121

S24) Oner-Iyidogan Y, Oner P, Kocak H, Gurdol F, Bekpinar S, Unlucerci Y, et al. Dimethylarginines and inflammation markers in patients with chronic kidney disease undergoing dialysis. Clin Exp Med. 2009; 9(3): 235-241, doi: 10.1007/s10238-009-0035-3

S25) Paiva H, Kahonen M, Lehtimaki T, Raitakari OT, Jula A, Viikari J, et al. Asymmetric dimethylarginine (ADMA) has a role in regulating systemic vascular tone in young healthy subjects: the cardiovascular risk in young Finns study. Am J Hypertens. 2008; 21(8): 873-878, doi: 10.1038/ajh.2008.215

S26) Paroni R, Fermo I, Fiorina P, Cighetti G. Determination of asymmetric and symmetric dimethylarginines in plasma of hyperhomocysteinemic subjects. Amino Acids. 2005; 28(4): 389-394, doi: 10.1007/s00726-005-0191-z

S27) Pascale V, Pascale W, Lavanga V, Sansone V, Ferrario P, De Gennaro Colonna V. L-arginine, asymmetric dimethylarginine, and symmetric dimethylarginine in plasma and synovial fluid of patients with knee osteoarthritis. Med Sci Monit. 2013; 19: 1057-1062, doi: 10.12659/MSM.889275

S28) Schwedhelm E, Xanthakis V, Maas R, Sullivan LM, Schulze F, Riederer U, et al. Asymmetric dimethylarginine reference intervals determined with liquid chromatography-tandem mass spectrometry: results from the Framingham offspring cohort. Clin Chem. 2009; 55(8): 1539-1545, doi: 10.1373/clinchem.2009.124263

S29) Selley ML. Increased (E)-4-hydroxy-2-nonenal and asymmetric dimethylarginine concentrations and decreased nitric oxide concentrations in the plasma of patients with major depression. J Affect Disord. 2004; 80(2-3): 249-256, doi: 10.1016/S0165-0327(03)00135-6

S30) Siroen MP, Teerlink T, Bolte AC, van Elburg RM, Richir MC, Nijveldt RJ, et al. No compensatory upregulation of placental dimethylarginine dimethylaminohydrolase activity in preeclampsia. Gynecol Obstet Invest. 2006; 62(1): 7-13, doi: 10.1159/000091752

S31) Surdacki A, Martens-Lobenhoffer J, Wloch A, Marewicz E, Rakowski T, Wieczorek-Surdacka E, et al. Elevated plasma asymmetric dimethyl-L-arginine levels are linked to endothelial progenitor cell depletion and carotid atherosclerosis in rheumatoid arthritis. Arthritis Rheum. 2007; 56(3): 809-819, doi: 10.1002/art.22424

S32) Szuba A, Chachaj A, Wrobel T, Dzietczenia J, Mazur G, Antonowicz-Juchniewicz J, et al.. Asymmetric dimethylarginine in hematological malignancies: a preliminary study. Leuk Lymphoma. 2008; 49(12): 2316-2320, doi: 10.1080/10428190802510323

S33) Szymczyszyn A, Doroszko A, Szahidewicz-Krupska E, Rola P, Gutherc R, Jasiczek J, et al. Effect of the transdermal low-level laser therapy on endothelial function. Lasers Med Sci. 2016. doi: 10.1007/s10103-016-1971-2

S34) Turiel M, Atzeni F, Tomasoni L, de Portu S, Delfino L, Bodini BD, et al. Non-invasive assessment of coronary flow reserve and ADMA levels: a casecontrol study of early rheumatoid arthritis patients. Rheumatology. 2009; 48(7): 834-839, doi: 10.1093/rheumatology/kep082

S35) Turiel M, Gianturco L, Ricci C, Sarzi-Puttini P, Tomasoni L, Colonna Vde G, et al. Silent cardiovascular involvement in patients with diffuse systemic sclerosis: a controlled cross-sectional study. Arthritis Care Res. (Hoboken) 2013; 65(2): 274-280, doi: 10.1002/acr.21819

S36) Turkcuoglu I, Engin-Ustun Y, Turan F, Kali Z, Karabulut AB, Meydanli M, et al. Evaluation of asymmetric dimethylarginine, nitric oxide levels and associated independent variables in obese and lean patients with polycystic ovarian syndrome. Gynecol Endocrinol. 2011; 27(9): 609-614, doi: 10.3109/09513590.2010.507291

S37) Verhoeven MO, Hemelaar M, Teerlink T, Kenemans P, van der Mooren MJ. Effects of intranasal versus oral hormone therapy on asymmetric dimethylarginine in healthy postmenopausal women: a randomized study. Atherosclerosis. 2007; 195(1): 181-188, doi: 10.1016/j.atherosclerosis.2006.09.018

S38) Verhoeven MO, Teerlink T, Kenemans P, Vogelvang TE, van der Mooren MJ, Group, H. M. R. R. Effects on asymmetric dimethylarginine of HMR 3339, a novel selective estrogen receptor modulator: a 12-week, randomized, placebo-controlled, double-blind, dose-ranging study in healthy postmenopausal women. Menopause. 2007; 14(2): 235-242, doi: 10.1097/01.gme.0000235367.47350.2a

S39) Xia W, Shao Y, Wang Y, Wang X, Chi Y. Asymmetric dimethylarginine and carotid atherosclerosis in Type 2 diabetes mellitus. J Endocrinol Invest. 2012; 35(9): 824-827, doi: 10.1007/BF03347101

S40) Xia W, Xu L, Xu W, Wang X, Yao Y. Asymmetric dimethylarginine is associated with carotid atherosclerosis in patients with essential hypertension. Clin Exp Hypertens. 2015; 37(5): 393-397, doi: 10.3109/10641963.2014.987393

S41) Yavuz Taslipinar M, Kilic N, Bayraktar N, Guler I, Gulcan Kurt Y, Goktas T, et al. Endothelial dysfunction and insulin resistance in young women with polycystic ovarian syndrome. Turk J Med Sci. 2014; 44(5): 787-791.

S42) Zincir S, Zincir SB, Doruk A, Erdem M, Celik C, Ak M, et al. Asymmetric dimethylarginine (ADMA) and treatment response relationship in male patients with first-episode schizophrenia: a controlled study. Psychiatry Res. 2014; 220(1-2): 76-80, doi: 10.1016/j.psychres.2014.07.013
