# Supplementary material for: The issue of plasma asymmetric dimethylarginine reference range – A systematic review and meta-analysis
Source: PLoS One. 2017 May 11;12(5):e0177493. doi: 10.1371/journal.pone.0177493 (PMC5426758; doi:10.1371/journal.pone.0177493)
Supplement: S2 Table — (DOC) [file pone.0177493.s003.doc]

| **Study** | **No. of participants** | **Manufacturer** | **Country** |
| --- | --- | --- | --- |
| Beltran 2014 (S1) | 23 | Immundiagnostik | Spain, Europe |
| Brenner 2012 (S2) | 30 | Immundiagnostik | Germany, Europe |
| Deneva 2011 (S3) | 150* | DLD Diagnostika | Bulgaria, Europe |
| Dogru 2012 (S4) | 35 | Immundiagnostik | Turkey, Europe |
| Dogru 2013 (S5) | 74 | Immundiagnostik | Turkey, Europe |
| El Shahawy 2015 (S6) | 20 | Immundiagnostik | Egypt, Africa |
| Ferroni 2008 (S7) | 25 | DLD Diagnostika | Italy, Europe |
| Fidan 2012 (S8) | 31 | Immundiagnostik | Turkey, Europe |
| Frieling 2012 (S9) | 32 | DLD Diagnostika | Germany, Europe |
| Kals 2007 (S10) | 63 | DLD Diagnostika | Estonia, Europe |
| Karadurmus 2012 (S11) | 37 | Immundiagnostik | Turkey, Europe |
| Madaric 2013 (S12) | 50 | Immundiagnostik | Slovakia, Europe |
| Petrica 2015 (S13) | 28 | Immundiagnostik | Romania, Europe |
| Santilli 2010 (S14) | 42 | DLD Diagnostika | Italy, Europe |
| Schulze 2005 (S15) | 500* | DLD Diagnostika | Germany, Europe |
| Sonmez 2010 (S16) | 30 | Immundiagnostik | Turkey, Europe |
| Sonmez 2015 (S17) | 70 | Immundiagnostik | Turkey, Europe |
| Sulicka 2012 (S18) | 20 | DLD Diagnostika | Poland, Europe |
| Surdacki 2012 (S19) | 40* | DLD Diagnostika | Poland, Europe |
| Telejko 2009 (S20) | 36 | DLD Diagnostika | Poland, Europe |
| Tuygun 2011 (S21) | 24 | DLD Diagnostika | Turkey, Europe |
| Uzun 2008 (S22) | 20 | DLD Diagnostika | Turkey, Europe |
| Wei 2012 (S23) | 28 | Schulze, 2004** | China, Asia |
| Yildizhan 2011 (S24) | 27 | Immundiagnostik | Turkey, Europe |

**Table 1** List of papers included in quantitative analysis using ELISA to determine ADMA concentrations.

* Subgroup data available

**The Authors used an ELISA method developed by Schulze et al. (2004) (S25)

S1) Beltran LM, Hernandez RM, Bernal RSD, Morillo JSG, Egido J, Noval ML, et al. Reduced sTWEAK and increased sCD163 levels in HIV-infected patients: modulation by antiretroviral treatment, HIV replication and HCV co-Infection. PLoS One. 2014; 9(3): e90541, doi:10.1371/journal.pone.0090541

S2) Brenner T, Fleming, TH, Rosenhagen C, Krauser U, Mieth M, Bruckner T, et al. L-arginine and asymmetric dimethylarginine are early predictors for survival in septic patients with acute liver failure. Mediators Inflamm. 2012; 2012: 210454, doi: 10.1155/2012/210454

S3) Deneva-Koycheva TI, Vladimirova-Kitova LG, Angelova EA, Tsvetkova TZ. Plasma asymmetric dimethylarginine levels in healthy people. Folia Med. (Plovdiv) 2011; 53(1): 28 – 33.

S4) Dogru T, Genc H, Tapan S, Ercin CN, Ors F, Aslan F. Elevated asymmetric dimethylarginine in plasma: an early marker for endothelial dysfunction in non-alcoholic fatty liver disease? Diabetes Res Clin Pract. 2012; 96(1): 47 – 52, doi: 10.1016/j.diabres.2011.11.022

S5) Dogru T, Genc H, Tapan S, Aslan F, Ercin CN, Ors F, et al. Plasma fetuin-A is associated with endothelial dysfunction and subclinical atherosclerosis in subjects with nonalcoholic fatty liver disease. Clin Endocrinol. (Oxf) 2013; 78(5): 712 – 717, doi: 10.1111/j.1365-2265.2012.04460.x

S6) El Shahawy Y, Soliman Y, Rifaie A, Shenawy H, Behairy M, Mady G. Relationship between asymmetric dimethylarginine plasma level and left ventricular mass in hemodialysis patients. Saudi J Kidney Dis Transpl. 2015; 26(1): 26 – 33.

S7) Ferroni P, Guagnano MT, Falco A, Paoletti V, Manigrasso MR, Michetti N. Association of low-grade inflammation and platelet activation in patients with hypertension with microalbuminuria. Clin Sci. (Lond) 2008; 114(6): 449 – 455, doi: 10.1042/CS20070307

S8) Fidan V, Alp H, Karaca F, Ozcan A, Ozcan K. Effect of endoscopic sinus surgery on plasma asymmetric dimethylarginine levels in patients with extensive nasal polyposis. J Int Med Res. 2012; 40(2): 565 – 571.

S9) Frieling H, Leitmeier V, Haschemi-Nassab M, Kornhuber J, Rhein M, Bleich S, et al. Reduced plasma levels of asymmetric di-methylarginine (ADMA) in patients with alcohol dependence normalize during withdrawal. Eur Neuropsychopharmacol. 2012; 22(11): 836 – 840, doi: 10.1016/j.euroneuro.2012.03.010

S10) Kals J, Kampus P, Kals M, Teesalu R, Zilmer K, Pulges A, et al. Arterial elasticity is associated with endothelial vasodilatory function and asymmetric dimethylarginine level in healthy subjects. Scand J Clin Lab Invest. 2007; 67(5): 536 – 544, doi: 10.1080/00365510701203470

S11) Karadurmus N, Tapan S, Cakar M, Naharci I, Celik T, Tasci I, et al. Lower plasma soluble TWEAK concentration in patients with newly diagnosed hypertension. Clinical and Investigative Medicine. 2012; 35(1): e20-e26.

S12) Madaric A, Kadrabova J, Krajcovicova-Kudlackova M, Valachovicova M, Spustova V, Mislanova C, et al. The effect of bioactive complex of quercetin, selenium, catechins and curcumin on cardiovascular risk markers in healthy population after a two month consumption. Bratisl Lek Listy. 2013; 114(2): 84 – 87.

S13) Petrica L, Vlad A, Gluhovschi G, Zamfir A, Popescu C, Gadalean F, et al. Glycated peptides are associated with proximal tubule dysfunction in type 2 diabetes mellitus. International Journal of Clinical and Experimental Medicine. 2015; 8(2): 2516 – 2525.

S14) Santilli F, Davi G, Basili S, Lattanzio S, Cavoni A, Guizzardi G, et al. Thromboxane and prostacyclin biosynthesis in heart failure of ischemic origin: effects of disease severity and aspirin treatment. J Thromb Haemost. 2010; 8(5): 914 – 922, doi: 10.1111/j.1538-7836.2010.03820.x

S15) Schulze F, Maas R, Freese R, Schwedhelm E, Silberhorn E, Boger RH. Determination of a reference value for N(G), N(G)-dimethyl-L-arginine in 500 subjects. Eur J Clin Invest. 2005; 35(10): 622 – 626, doi: 10.1111/j.1365-2362.2005.01561.x

S16) Sonmez A, Celebi G, Erdem G, Tapan S, Genc H, Tasci I, et al. Plasma Apelin and ADMA Levels in Patients with Essential Hypertension. Clinical and Experimental Hypertension. 2010; 32(3): 179 – 183, doi: 10.3109/10641960903254505

S17) Sonmez A, Haymana C, Aydogdu A, Tapan S, Basaran Y, Meric C, et al. Endothelial dysfunction, insulin resistance and inflammation in congenital hypogonadism, and the effect of testosterone replacement. Endocr J. 2015; 62(7): 605 – 613, doi: 10.1507/endocrj.EJ15-0125

S18) Sulicka J, Surdacki A, Strach M, Kwater A, Gryglewska B, Cwiklinska M, et al. Elevated asymmetric dimethylarginine in young adult survivors of childhood acute lymphoblastic leukemia: a preliminary report. Dis Markers. 2012; 33(2): 69 – 76, doi: 10.3233/DMA-2012-0906

S19) Surdacki A, Zawislak B, Rakowski T, Wieczorek-Surdacka E, Fedak D, Dubiel JS. Parental history of premature coronary artery disease does not affect plasma levels of asymmetric dimethylarginine in young healthy adults. Pol Arch Med Wewn. 2012; 122(10): 487 – 493.

S20) Telejko B, Zonenberg A, Kuzmicki M, Modzelewska A, Niedziolko-Bagniuk K, Ponurkiewicz A, et al. Circulating asymmetric dimethylarginine, endothelin-1 and cell adhesion molecules in women with gestational diabetes. Acta Diabetol. 2009; 46(4): 303 – 308, doi: 10.1007/s00592-008-0088-x

S21) Tuygun AK, Tuygun A, Yurtseven N, Sensoz Y, Gunay R, Keser M, et al. Asymmetric dimethylarginine levels in Buerger's disease. Ann Vasc Surg. 2011; 25(4): 547 – 554, doi: 10.1016/j.avsg.2010.12.011

S22) Uzun H, Konukoglu D, Besler M, Erdenen F, Sezgin C, Muderrisoglu C. The effects of renal replacement therapy on plasma, asymmetric dimethylarginine, nitric oxide and C-reactive protein levels. Clin Invest Med. 2008; 31(1): e1-e7.

S23) Wei, D, He WY, Lv QZ. Effect of nisoldipine and olmesartan on endothelium-dependent vasodilation in essential hypertensive patients. CNS Neurosci Ther. 2012; 18(5): 400 – 405, doi: 10.1111/j.1755-5949.2012.00304.x

S24) Yildizhan R, Ilhan GA, Yildizhan B, Kolusari A, Adali E, Bugdayci G. Serum retinol-binding protein 4, leptin, and plasma asymmetric dimethylarginine levels in obese and nonobese young women with polycystic ovary syndrome. Fertil Steril. 2011; 96(1): 246 – 250, doi: 10.1016/j.fertnstert.2011.04.073

S25) Schulze F, Wesemann R, Schwedhelm E, Sydow K, Albsmeier J, Cooke JP, et al. Determination of asymmetric dimethylarginine (ADMA) using a novel ELISA assay. Clin Chem Lab Med. 2004; 42(12): 1377**–**1383.
